# Host Lipid Bodies as Platforms for Intracellular Survival of Protozoan Parasites

**DOI:** 10.3389/fimmu.2016.00174

**Published:** 2016-05-03

**Authors:** Daniel A. M. Toledo, Heloísa D’Avila, Rossana C. N. Melo

**Affiliations:** ^1^Laboratory of Cellular Biology, Department of Biology, Institute of Biological Sciences (ICB), Federal University of Juiz de Fora (UFJF), Juiz de Fora, Minas Gerais, Brazil

**Keywords:** infectious diseases, lipid droplets, inflammation, phagocytosis, lipid mediators, parasite survival, parasitophorous vacuole

## Abstract

Pathogens induce several changes in the host cell signaling and trafficking mechanisms in order to evade and manipulate the immune response. One prominent pathogen-mediated change is the formation of lipid-rich organelles, termed lipid bodies (LBs) or lipid droplets, in the host cell cytoplasm. Protozoan parasites, which contribute expressively to the burden of infectious diseases worldwide, are able to induce LB genesis in non-immune and immune cells, mainly macrophages, key players in the initial resistance to the infection. Under host–parasite interaction, LBs not only accumulate in the host cytoplasm but also relocate around and move into parasitophorous vacuoles. There is increasing evidence that protozoan parasites may target host-derived LBs either for gaining nutrients or for escaping the host immune response. Newly formed, parasite-induced LBs may serve as lipid sources for parasite growth and also produce inflammatory mediators that potentially act in the host immune response deactivation. In this mini review, we summarize current knowledge on the formation and role of host LBs as sites exploited by intracellular protozoan parasites as a strategy to maintain their own survival.

## Introduction

Protozoan parasitic infections comprise devastating infectious diseases, such as malaria, visceral leishmaniasis, toxoplasmic encephalitis, and trypanosomiasis, which still account for a large proportion of death and disability worldwide (1). Many protozoan parasites have an obligate intracellular existence. The infection is initiated when the parasite enter the host target cell and is internalized within a plasma membrane-derived vacuole, the parasitophorous vacuole (PV) (2). Within the host, protozoan parasites that are mainly intracellular will only cause infectious disease if they are able to survive and multiply within the PV (2, 3).

The events of the PV formation and progression generally occur in parallel with accentuated genesis of lipid-rich organelles, termed lipid bodies (LBs) or lipid droplets, in the host cell cytoplasm [reviewed in Ref. (4, 5)]. It is now well documented that experimental and clinical infections with a range of protozoan parasites trigger LB accumulation (Table 1) and an intriguing interaction with the PV [reviewed in Ref. (5)]. Other pathogens, such as bacteria and viruses, also induce LB formation within different cell types, indicating that LB accumulation in response to infectious diseases is a broad event and may have implications for microbial pathogenesis (5–8).

**Table 1:** **Protozoan parasite-induced lipid body formation in host cells**.

Parasite	Cell type	Organism/model	Reference
*Leishmania amazonensis*	Peritoneal macrophages	Mouse	(9)
	Dendritic leukocytes	Mouse	(10)
*Leishmania major*	Blood-marrow-derived macrophages	Mouse	(11, 12)
*Plasmodium berghei*	Hepatocytes	Mouse	(13)
	Renal tubular cells	Mouse	(14)
*Plasmodium chabaudi*	Hepatocytes	Mouse	(15)
*Toxoplasma gondii*	Fibroblasts	Human	(16)
	Skeletal muscle cells	Mouse	(17)
*Trypanosoma cruzi*	Heart macrophages	Rat	(18, 19)
	Peritoneal macrophages	Rat	(18, 19)
	Uterine macrophages	Rat	(19)
	Peritoneal macrophages	Mouse	(20)
	Placental cells	Human	(21)

While the successful replication within the PV is under influence of several factors, there is increasing evidence that LB organelles are important for the rapid intracellular reproduction of protozoan parasites (5). Protozoan parasites require large amounts of lipids necessary for membrane biogenesis of new progenies [reviewed in Ref. (22, 23)] and may take advantage of these organelles as high-energy substrate sources. LBs within infected cells are also able to produce inflammatory mediators that potentially can inhibit the host Th1response, thus, favoring parasite growth [reviewed in Ref. (4)]. In this mini review, we will discuss the role of host LBs as organelles modulated by intracellular protozoan parasites for survival.

## LB Structure and Visualization

Lipid bodies are common organelles distributed in the cytoplasm of prokaryotic and eukaryotic cells (5). As a general feature, LBs contain a core rich in neutral lipids, surrounded by a monolayer of phospholipids with structural proteins – the perilipin (PLIN) family proteins (5). Proteins are not restricted to the LB surface. It is documented that many types of proteins are present in the LB internum depending on the cell type (24–26).

**Figure 1:**
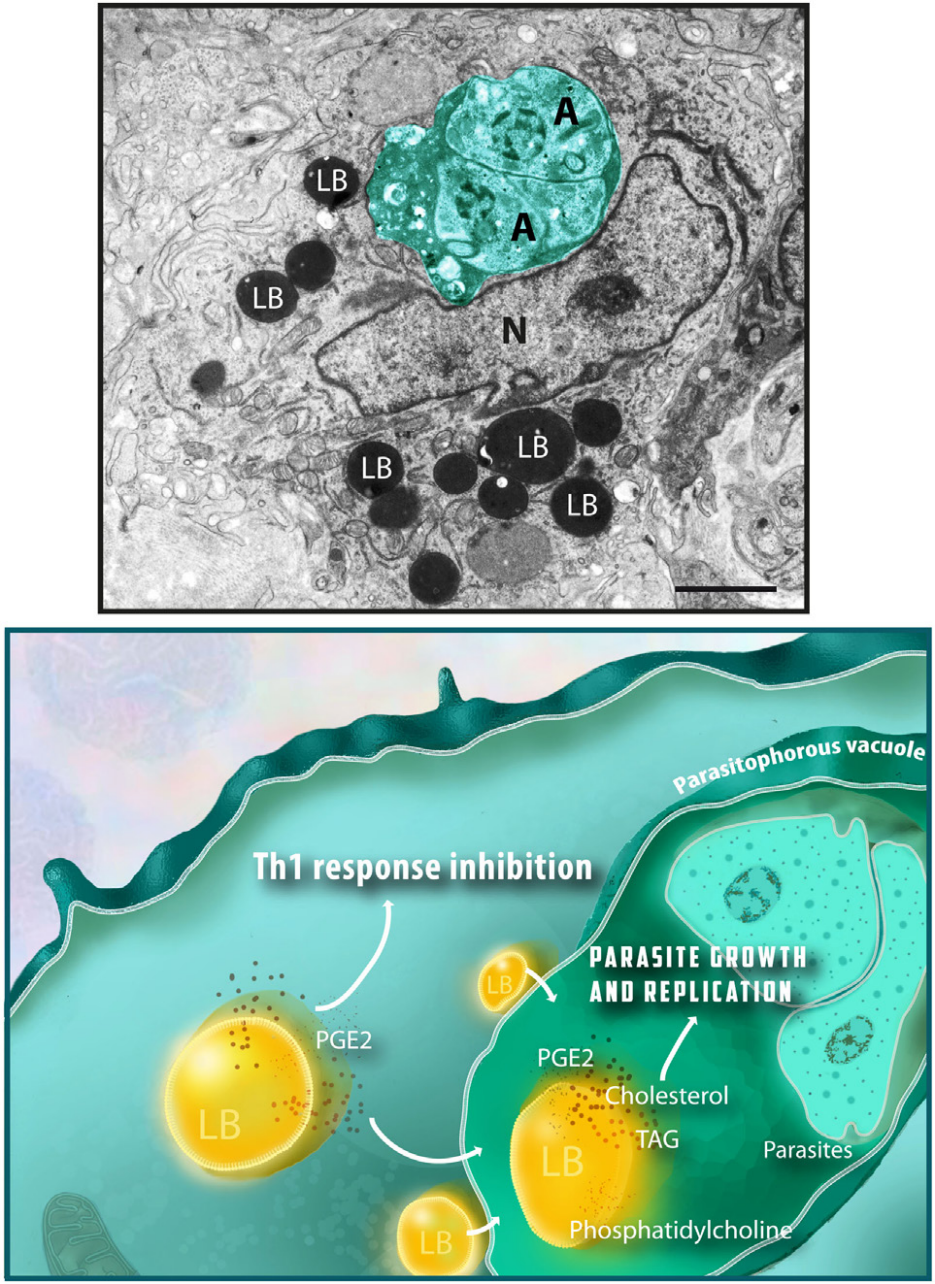
**Lipid bodies (LBs) accumulate in the host cell cytoplasm in response to interaction with protozoan parasites and favor parasite survival**. Top panel shows an electron micrograph of a heart inflammatory macrophage infected with *Trypanosoma cruzi*. Note the presence of dividing amastigotes (A) within the parasitophorous vacuole (outlined in green) and the high number of LBs in the cytoplasm. LBs typically appear as round electron dense organelles in the cell cytoplasm. A model to explain how protozoan parasite-induced LB formation favors parasite growth and replication is shown in the bottom panel. Interaction of LBs with the parasitophorous vacuole, an event also triggered by the parasite infection, leads to discharge of LB contents, such as cholesterol, triacylglycerol (TAG), and phosphatidylcholine, which serve as lipid sources for parasite growth. Newly formed LBs are also sites for prostaglandin E2 synthesis (PGE2), a potent inflammation mediator that potentially inhibit the host Th1 immune response, thus decreasing the microbicidal capacity of the parasitophorous vacuole. Nu, nucleus. Scale bar, 600 nm. Top panel was reprinted from Ref. (27) with permission.

In spite of variations in the LB composition, these organelles are seen in the cell cytoplasm as compartmentalized round sites by light microscopy or transmission electron microscopy (TEM). While LB imaging under light microscopy usually requires the use of specific lipid probes, ultrastructural observation does not need any additional labeling because LBs lack a true delimiting unit membrane structure, which enables unambiguous identification by TEM (Figure 1) (4, 8, 26).

## Protozoan Parasites Induce LB Accumulation in Host Cells

Several protozoan parasites induce LB formation in a variety of host immune and non-immune cells (Table 1). Under host–parasite interaction, LBs not only accumulate but also increase in size and undergo ultrastructural changes in the host cell cytoplasm. These structural modifications of LBs have been well demonstrated during *in vitro* and *in vivo* studies with *Trypanosoma cruzi*, the causal agent of Chagas’ disease (18, 19). This pathology elicits a strong inflammatory response characterized by elevated infiltration of macrophages in the target organs, mainly the heart (28). Histopathological studies conducted during the acute phase of this disease showed that increased myocardial parasitism is paralleled by increased size of LBs within inflammatory macrophages in which LBs can reach up to 4 μm in diameter (19). Moreover, LBs show varied electron-density in response to the *T. cruzi* infection, a morphological change likely associated with mobilization and/or *in situ* synthesis of lipid mediators [reviewed in Ref. (8)]. The capability of host LBs to generate these compounds will be discussed later.

Increase of LB size in parallel with augmented number of these organelles was also found in dendritic leukocytes hosting live *Leishmania amazonensis* amastigotes (10). Overall, as noted in diverse cell types (Table 1), it is clear that protozoan parasites are able to trigger LB formation in host cells. But how is the mechanism leading to LB genesis? Similar to other studies with mycobacteria (29, 30), it has been demonstrated that parasite uptake potentiates LB formation within host cells but is not essential for triggering this event. After 24 h of infection with *T. cruzi*, peritoneal macrophages with internalized parasites as well as non-parasitized cells show increased number of LBs compared to control, non-infected cells, suggesting a bystander amplification of the cell response (20). Accordingly, inhibition of parasite phagocytosis did not abolish LB genesis during infection with *Leishmania major* (11). These authors have recently showed that while phagocytosis of latex beads by macrophages did not trigger LB formation, this phenomenon is equally induced by both live and heat-killed parasites (11). Altogether, these results imply that soluble factors in parasitized cells may act in a paracrine manner to produce LB in non-parasitized bystander cells (11). While LB formation occurs through a toll-like receptor-2 (TLR2)-dependent mechanism as documented during *T. cruzi* infection in macrophages (20), the identification of downstream signaling pathways involved in this event during parasitic infections awaits further investigation.

## Newly Formed LBs are Recruited to the Proximity of Parasites

Interestingly, protozoan parasites trigger a redistribution of the newly formed LBs around parasite-containing phagosomes. As documented by studies with *T. cruzi* (18, 19), *L. major* (11, 12), *L. amazonensis* (10), and *Toxoplasma gondii* (17, 31), LBs accumulate in close proximity to PVs or even move into these vacuoles, suggesting that these pathogens take advantage of these organelles.

Indeed, TEM analyses have enabled the identification of intimate contact between LBs and the PV membrane (10, 17–20, 31, 32). In 2003, our group was the first to observe that during an *in vivo* experimental infection with a pathogen (*T. cruzi*), LBs were internalized into parasite-containing phagosomes (18). Following studies found the same event for infections with different species of bacteria [reviewed in Ref. (5)] and with the protozoan parasites *T. gondii* (17) and *L. major* (11).

How LBs translocate across the phagosome membrane? This mechanism has been mainly addressed in studies with bacteria. LB-associated proteins, secreted by bacteria seem to be involved in capturing LB into bacteria-containing vacuoles, while LB translocation seems to involve displacement of the LB structural protein PLIN2/adipose differentiation-related protein (ADRP), which is constitutively associated with the surface of LBs (33). Although studies of membrane sites between pathogen-containing compartments and intracellular host organelles has been gaining more attention in the literature [reviewed in Ref. (34)], the mechanistic details underlying the intriguing LB–phagosome interaction remains to be fully defined.

## Host LIPIDS, LBs, and Intracellular Parasite Growth

Lipid metabolic pathways have been demonstrated as major networks modulated by protozoan parasites in host cells. Numerous studies with *T. gondii*, malaria parasites (*Plasmodium berghei, Plasmodium falciparum*, *and Plasmodium yoelii)*, *L. amazonensis*, *Cryptosporidium parvum*, and *Eimeria bovis* have documented that these parasites are able to (i) induce accumulation of lipids, such as neutral lipids [triacylglycerol (TAG), diacylglycerol, and cholesterol esters], cholesterol, and/or phospholipids, especially phosphatidylcholine in host cells (10, 12, 35–37); (ii) acquire lipid resources, such as cholesterol and phospholipids from their host environment into the PV (16, 36, 38–40), and/or (iii) use host lipids to synthesize complex own lipids or even own LBs (16, 38, 41).

Thus, although the nutritional requirements of these parasites are intricate, overall it is believed that host lipids are central to support successful parasite replication within the PV (16, 32, 37, 38, 42, 43). Host lipid acquisition is also considered crucial to PV maturation. This vacuole undergoes a pronounced membranous remodeling associated with formation of an internal network of tubules and vesicles as observed during the infection with *T. gondii* (42, 44). By using different approaches, including fluorescence recovery after photobleaching (FRAP) microscopy, Caffaro and Boothroyd demonstrated that host cells are major lipid contributors to the PV remodeling and that lipids are transferred in a continuous way from the host into the PV (42).

How is the contribution of host LBs as lipid sources for protozoan parasites? Consistent with parasite-induced host LB formation (Table 1), LB genesis in parallel to host lipid reprograming/accumulation (10, 12) and host LB–PV interaction (10–12, 17–19, 31), it is believed that these organelles act as important lipid sources for parasite growth.

Several classes of lipids, including neutral lipids, cholesterol, and phospholipids, make up LBs (45). Because protozoan parasites are not competent or have limited ability to synthesize lipids [reviewed in Ref. (22, 46)], LBs could be an essential source of both TAG and cholesterol for these parasites (10). These molecules are important as precursors for membrane neogenesis for newly formed parasites [reviewed in Ref. (22, 46)]. Moreover, the phospholipid monolayer of LBs consists of numerous phospholipid species of which phosphatidylcholine is the most abundant (47). Thus, it is likely that LB-derived lipids are transferred from host to the PV and taken up by replicating parasites. Indeed, by using a fluorescent probe (BODIPY-phosphatidylcholine) and live imaging microscopy, Charron and Sibley showed that this probe moved from host plasma membrane and/or host LBs, seen as dispersed puncta in the host cytoplasm, to LBs formed within the parasite (16). However, the potential relocation of other lipids from host LBs to the parasite still needs to be better explored in future studies.

Does depletion of host lipids and/or LBs impact protozoan parasites development within host cells? In *T. gondii* infection, inhibition of cholesterol esterification in the host cell blocks parasite growth (38). Exposure to cholesterol esterification inhibitors led to reduction of cholesteryl ester synthesis, morphological changes in parasite LBs, and deformations of the parasite plasma membrane with discharge of parasite content into the PV (38). Accordingly, the use of inhibitors targeting the host cellular cholesterol *de novo* synthesis and processing repressed both *E. bovis* proliferation and LB formation within host endothelial cells (43). On the other hand, depletion of cholesterol content did not impact malaria parasites (*P. berghei and P. yoelii*) development within hepatocytes (39). In this case, the authors consider that the parasite may exploit alternative sources in these cells to sustain infectivity (39). In fact, protozoan parasites can divert lipids from other sources than the intracellular environment. *C. parvum*, the causal agent of cryptosporidiosis, a life-threatening diarrheal disease in immunocompromised individuals (48), scavenges cholesterol from plasma low-density protein (LDL) and micelles, and to a lesser extent from the cholesterol pathway within enterocytes (40). Removal of cholesterol from the media, and to lesser extent from host intracellular pools, obstructs parasite reproduction (40).

During the experimental infection with *T. cruzi*, our group demonstrated that the use of C75, an inhibitor of fatty acid synthase, led to both inhibition of LB formation and parasite division within macrophages (20). While C75 had no direct cytotoxic effect on the parasite, intracellular parasite replication was likely affected by an accentuated reduction of the LB numbers (around 66%) after cell treatment with C75 (20).

## Host LBs Produce PGE2 in Response to Infection with Protozoan Parasites

One interesting aspect of LBs is that they are able to change their composition in response to inflammatory events as documented in cells from the immune system [reviewed in Ref. (49)]. LBs contain stores of arachidonic acid (AA), indicating that these organelles are potentially able to initiate cascades that culminate in the formation of inflammatory mediators (eicosanoids) (50, 51). Eicosanoid-generating enzymes (52–55), and *in situ* synthesis of eicosanoids (prostaglandins and leukotrienes) were indeed documented in these organelles within activated leukocytes and other cells from the immune system during inflammatory conditions [for example, see Ref. (29, 56–58)].

During the infection with *T. cruzi* (18), *T. gondii* (17, 31), and *L. amazonensis* (9), significant correlations between LB formation and enhanced generation of eicosanoids, specifically prostaglandin E2 (PGE2), by host cells have been observed. Moreover, *T. gondii* infection also elicited a time-dependent increase of cyclooxygenase-2 (COX-2) mRNA levels, indicating that the PGE2 may be a product of an active COX pathway within host cells (17).

By investigating the intracellular specific localization of both COX-2 and PGE2 within *T. cruzi*-infected macrophages, we found that both molecules were immunolocalized in LBs, indicating that LBs act as sources of PGE2 (20). Interestingly, cell treatment with non-steroidal anti-inflammatory drugs (NSAIDs) inhibited both LB formation and LB-derived PGE2 synthesis in a mechanism independent of COX inhibition (20), as previously documented (59). Therefore, accumulation of LBs in infected host cells may modulate the production of an innate immune response with production of PGE2, which in turn may contribute to a permissive environment for pathogen proliferation. For example, during the infection with *T. gondii*, the increased numbers of LBs within macrophages correlated with high PGE2 levels, decreased nitric oxide (NO) production and parasite survival (31). In fact, high concentrations of PGE2 potentially inhibit the Th1 response, tumor necrosis factor alpha (TNF-α) and/or NO production (60–62). This scenario is also detected during infections with mycobacteria (29, 30), demonstrating that pathogen-induced LB formation associated with PGE2 synthesis is a broader event that can potentially support intracellular pathogen survival. Taken together, these data suggest a model by which LBs are acting as potential stations for the survival of protozoan parasites within host cells, as depicted in Figure 1.

## Final Remarks and Future Directions

Several key issues remain to be addressed to better understand the link between LBs and parasite survival within host cells during infections with intracellular parasitic protozoans. It is now clear that host LBs interact with PVs and that the parasite has a remarkable ability to sequester host lipids. What are the molecular mechanisms involved in LB translocation into the PV and how the LB content is extracted by the parasite? In patients with chronic mycobacterial infection (*Mycobacterium leprae*), round classical LBs are observed in contact with pathogen-containing phagosomes and intact bacteria are seen completely enmeshed in accumulated lipid content within the vacuole (63). Are LBs important for pathogen survival in chronic parasitic infections? Considering that LBs are sources for parasite development, could these organelles be target by therapeutic treatment? How significant is the contribution of host LBs and/or parasite LBs to parasite development? Moreover, the mechanisms by which new molecules, including natural products, can affect pathways of the parasite lipid metabolism and both host and parasite LB formation have yet to be fully appreciated.

## Author Contributions

RM prepared the manuscript. All authors contributed in part to writing and editing the manuscript and approved the final version.

## Conflict of Interest Statement

The authors declare that the research was conducted in the absence of any commercial or financial relationships that could be construed as a potential conflict of interest.
